# Cytokine and Chemokine Profiling in Patients with Hand, Foot and Mouth Disease in Singapore and Malaysia

**DOI:** 10.1038/s41598-018-22379-6

**Published:** 2018-03-06

**Authors:** Fiona Mei Shan Teo, Min Nyo, Anng Anng Wong, Natalie Woon Hui Tan, Mia Tuang Koh, Yoke Fun Chan, Chia Yin Chong, Justin Jang Hann Chu

**Affiliations:** 1 Collaborative and Translation Unit for HFMD, Institute of Molecular and Cell Biology, Agency for Science, Technology and Research (A*STAR), Singapore, Singapore; 2 Laboratory of Molecular RNA Virology and Antiviral Strategies, Department of Microbiology and Immunology, Yong Loo Lin School of Medicine, National University of Singapore, Singapore, Singapore; 3 Infectious Disease Service, Department of Pediatrics, KK Women’s and Children’s Hospital, Singapore, Singapore; 4 Department of Pediatrics, Faculty of Medicine, University of Malaya, Kuala Lumpur, Malaysia; 5 Department of Medical Microbiology, Faculty of Medicine, University of Malaya, Kuala Lumpur, Malaysia

## Abstract

Hand, foot and mouth disease (HFMD) is a prevalent contagious childhood disease typically associated with fever, oral lesions and limb exanthema. While HFMD is caused by a plethora of serotypes of viruses under the genus *Enterovirus* within the *Picornaviridae* family, Coxsackievirus A16 (CV-A16) and Enterovirus 71 (EV-A71) are considered the main etiological agents. In recent years however, other viruses have also been isolated in considerable numbers from infected individuals in many regions, joining the legion commonly associated with HFMD. The present study investigated the cytokine and chemokine profiles of HFMD patients from Singapore and Malaysia for the first time. Comparative cohort studies of EV-A71-associated HFMD cases revealed that the Malaysia cohort had a distinct profile from the Singapore cohort, and this could be partly attributed by different EV-A71 genotypes. As the isolation of CV-A6, instead of CV-A16, had become prevalent in the Singapore cohort, it was also of particular interest to study the differential cytokine and chemokine profiles. Our data revealed that overlapping as well as unique profiles exist between the two major causative clinical isolates in the Singapore cohort. Having a better understanding of the respective immunological profiles could be useful for more accurate HFMD diagnosis, which is imperative for disease transmission control until multi-valent vaccines and/or broad-spectrum anti-viral drugs become available.

## Introduction

Hand, foot and mouth disease (HFMD) is a widespread transmissible infectious disease caused by a myriad of etiological agents under the genus *Enterovirus* within the *Picornaviridae* family, with Coxsackievirus A16 (CV-A16) and Enterovirus A71 (EV-A71) generally regarded as its major causative agents^1^. CV-A16 is regarded as the most dominant serotype that causes asymptomatic or mild HFMD which resolves on its own most of the time. EV-A71, on the other hand, is a neurotropic virus associated with neurological complications and even deaths in infants and children in the Asia Pacific region^2–7^. Due to its virulence, EV-A71 causes a raising concern in the otherwise generally mild and self-limiting disease. Over the past two decades, tremendous research has been done to compare these two key players.

In addition to CV-A16 and EV-A71, other serotypes such as CV-A4, CV-A5, CV-A6, CV-A7, CV-A9, CV-A10, CV-A24, Coxsackievirus B2 (CV-B2), CV-B3, CV-B4, CV-B5, EV-G18, EV-D70 and Echovirus 7 (E-7) are also known to cause HFMD albeit in smaller numbers^3,8^. Some of these serotypes, however, are becoming more prevalent in the recent years, being capable of existing as the main circulating virus of HFMD outbreaks in some regions.

In particular, CV-A6 is an emerging HFMD-causing virus strain capable of causing outbreaks in many regions^9–18^. In the recent years, the two predominant strains causing HFMD outbreaks in Singapore have also shifted from the conventional CV-A16 and EV-A71 to CV-A6 and EV-A71^19–23^. Similar to CV-A16, CV-A6 infections are usually self-limiting unlike neurotrophic EV-A71 infections. However, CV-A6 infections are reportedly associated with atypical clinical presentations including onychomadesis^9–11,14,15^, eczema herpeticum^17^, skin rashes and/or eruptions at unusual sites^14,18^, varicella-like skin eruptions^18^, allergic dermatitis-like rashes^12^ and desquamation of palms and soles^10,14,18^. As the paradigm changes, it is imperative that a better understanding of CV-A6, the emerging HFMD causative agent, is achieved.

This study was aimed at analysing the cytokine and chemokine profiles of HFMD patients from Singapore and Malaysia. Cytokines are a group of small secretory signalling molecules with diverse immune-related roles^24^. Chemokines are a subclass of cytokines which have chemotactic properties^25^. Several independent studies had previously shown an association between elevated inflammatory cytokines and HFMD pathogenesis and progression^26–39^. However, none of these studies has examined HFMD cases from Singapore and Malaysia. In addition, previous studies have not analysed HFMD cases associated with CV-A6 infections, which was addressed in the present study.

## Results and Discussion

The recruited HFMD study cohort was screened for its viral etiology and the individuals were grouped according to the causative etiological agents that they were infected with. There were a total of 2 CV-A16-infected patients, 11 EV-A71-infected patients, 10 CV-A6-infected patients, and 9 healthy volunteers enrolled for the Singapore cohort, as well as 1 CV-A16-infected patient, 34 EV-A71-infected patients, and 1 CV-A6-infected patient enrolled for the Malaysia cohort. Since this study involved children of young age, it was difficult to obtain parental consent and voluntary compliance in the collection of sera from the HFMD patients and healthy children in Singapore/Malaysia’s context. Hence, we were unable to enrol a large cohort for our studies.

While EV-A71 remained as the major serotype isolated in both the Singapore and Malaysia cohorts, we observed a paradigmatic shift towards the CV-A6 serotype in the more recent recruitment from Singapore. On the contrary, the supposedly common CV-A16 serotype was rarely isolated in both our study cohorts. Several studies also reported similar epidemiological trends^40^.

In this retrospective study, we focused on evaluating the cytokine and chemokine responses of EV-A71-associated HFMD cases between the Singapore and Malaysia cohort. In addition, we also explored the cytokine and chemokine responses of HFMD patients infected by EV-A71 and CV-A6 from Singapore. To our knowledge, cytokine and chemokine profiling has not been done in CV-A6-associated HFMD cases, which is of our interest to its recent predominance in Singapore. The fine-tuning of the levels of myriad cytokines and chemokines, which are crucial immune modulators, upon viral infection determines an anti-viral state advantageous to the hosts or a pro-viral state advantageous to the invading viruses. Hence, evaluation of the cytokines and chemokines would provide insights to the dynamics of HFMD disease development depending on the causative agents implicated. We profiled the HFMD and healthy control sera for an array of 48 cytokines and chemokines and compared the levels across the different serotypes (Supplementary Table S1). CV-A16-infected patients from both cohorts and CV-A6-infected patients from the Malaysia cohort were excluded from the comparative analyses as the extremely small sample size is not a true representative of the respective serotypes.

**Figure 1:**
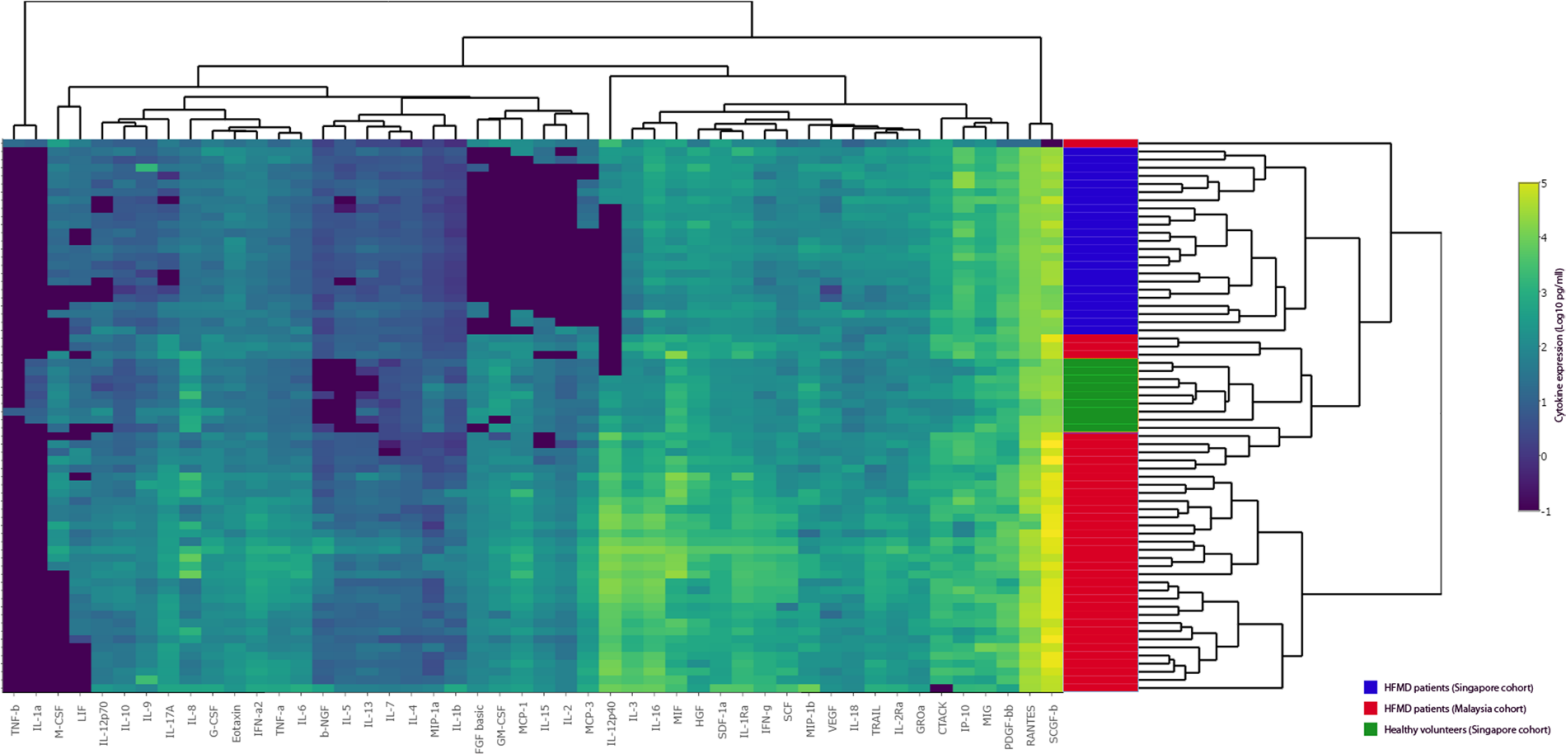
Heat map comparing the sera cytokine and chemokine expression profiles of HFMD patients revealed two distinctive clusters representing HFMD patients from Singapore and Malaysia respectively.

The heat map shown in Fig. 1 revealed two distinctive clusters representing HFMD patients from the two different cohorts, implying dissimilarities in the dysregulation of cytokines/chemokines between the Singapore and Malaysia cohorts.

**Figure 2:**
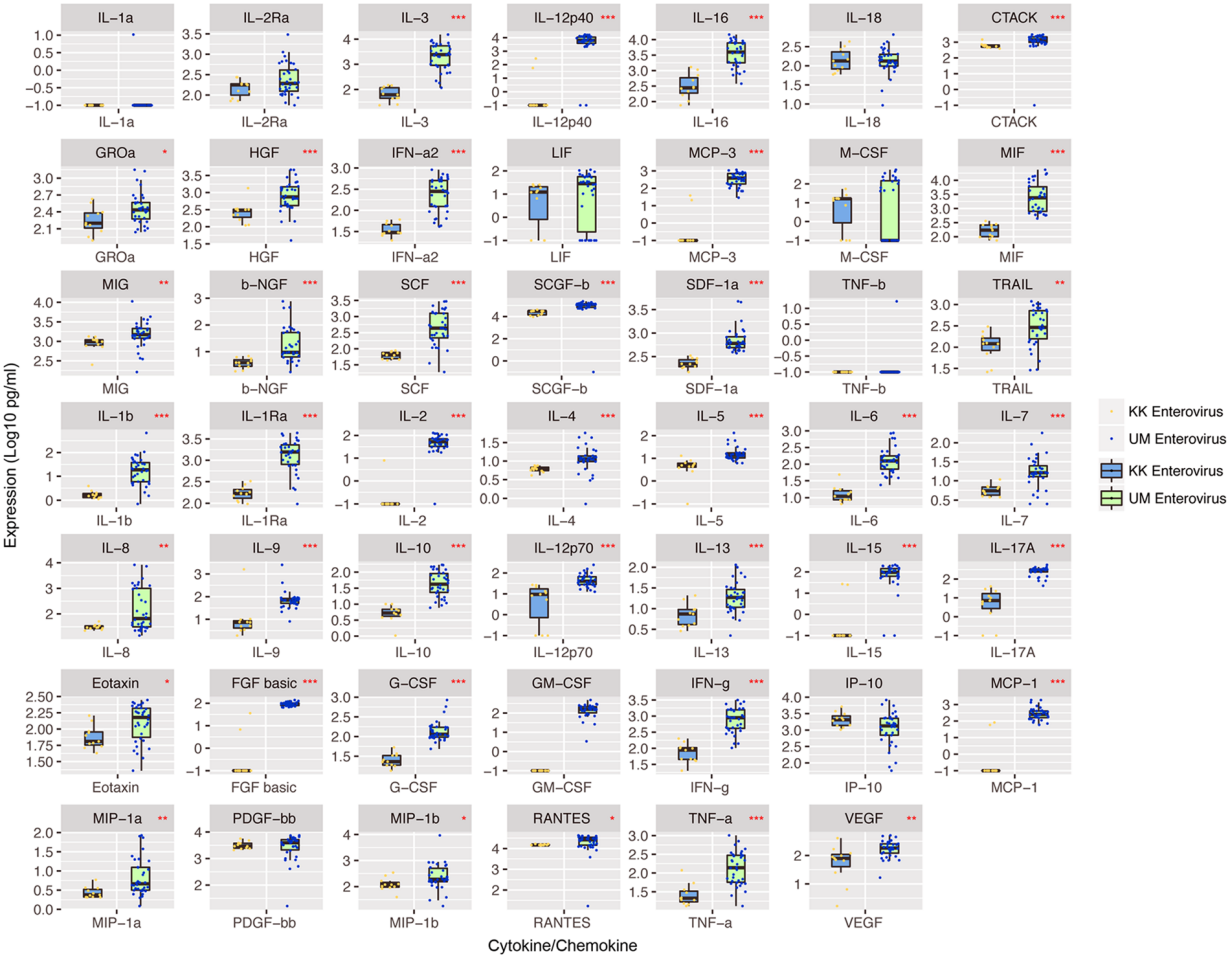
The expression profiles of EV-A71-infected patients from Singapore and Malaysia showed significant differences in 39 out of the panel of 48 cytokines and chemokines assayed. The statistical analyses were performed using GraphPad Prism version 4.0 (GraphPad software, USA), using Mann-Whitney non-parametric tests (without Gaussian distribution assumption). *p value < 0.05, **p value < 0.01, ***p value < 0.001. KK Enterovirus refers to EV-A71 cases from Singapore cohort, while UM Enterovirus refers to EV-A71 cases from Malaysia cohort.

EV-A71 serotypes were compared between the two cohorts, and Mann-Whitney non-parametric tests confirmed statistically significant differences between the two cohorts in 39 out of the panel of 48 cytokines and chemokines assayed (Fig. 2).

Since the Malaysia cohort was recruited in 2000 and 2012 while the Singapore cohort was recruited between 2013 and 2014, we speculated that the circulating EV-A71 (the major serotype isolated) genotype might be a contributing factor to the differences observed. To address this, comparisons were made between the EV-A71-infected patients recruited in 2000 and those recruited in 2012, where the main circulating EV-A71 genotypes were B4/C5 and B5 respectively^41^. Mann-Whitney non-parametric tests showed that 13 out of 48 cytokines were statistically different between the two periods of recruitment, where the HFMD patients from 2000 had higher sera levels of these cytokines/chemokines except for CTACK (Supplementary Figure S2). This might suggest that different EV-A71 genotypes could trigger host immune responses in different magnitudes, thereby emphasising a need to consider the circulating genotype when studying cytokine/chemokine dysregulation in EV-A71-associated HFMD.

**Figure 3:**
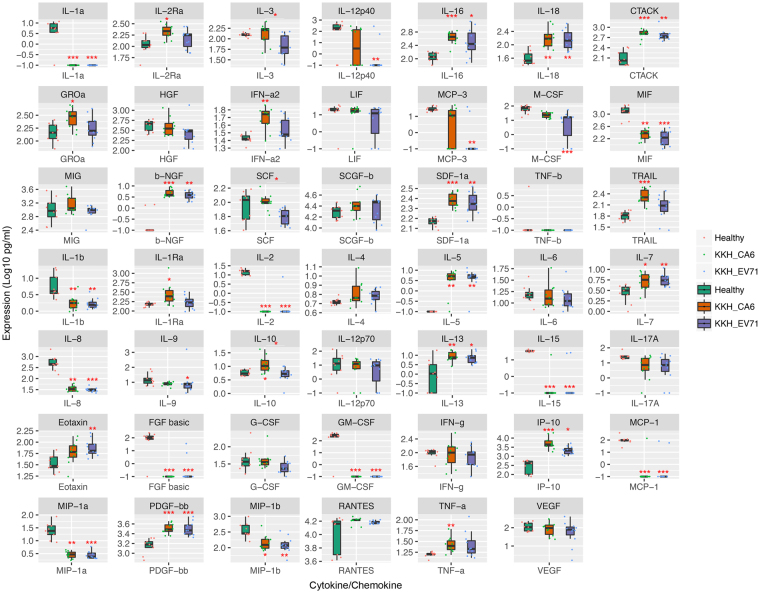
The cytokine and chemokine expression profiles of HFMD patients from Singapore showed significant differences between the two major etiological agents they were infected with. The statistical analyses were performed using GraphPad Prism version 4.0 (GraphPad software, USA), using Kruskal-Wallis non-parametric test (without Gaussian distribution assumption) coupled with Dunns post-test. All p-values were automatically adjusted by the program to account for false discovery rates associated with multiple comparisons. *p value < 0.05, **p value < 0.01, ***p value < 0.001. All p values stated were referenced to the healthy cohort, unless otherwise indicated by lines. Healthy refers to healthy volunteers, KKH_CA6 refers to CV-A6 cases, and KKH_EV71 refers to EV-A71 cases from Singapore cohort.

Previous profiling studies mainly explored HFMD or HFMD of different severities in EV-A71- and/or CV-A16- infected patients^26–39^. Since CV-A6-associated HFMD cases were becoming more prevalent in the Singapore cohort, we were particularly interested in its differential cytokine/chemokine dysregulation in comparison to EV-A71-associated HFMD cases since this area had not been explored. A comparative analysis was performed between EV-A71- and CV-A6-associated HFMD cases in the Singapore cohort, using healthy control sera as a reference (Fig. 3).

Interestingly, EV-A71 and CV-A6 shared a large number of cytokines/chemokines dysregulation: EV-A71 and CV-A6-infected HFMD patients had significantly reduced levels of sera IL-1α, MIF, IL-1β, IL-2, IL-8, IL-15, FGF-basic, GM-CSF, MCP-1, MIP-1α, and MIP-1β as well as increased levels of sera IL-16, IL-18, CTACK, β-NGF, SDF-1α, IL-5, IL-7, IL-13, IP-10, and PDGF-ββ. In addition to the large set of commonly dysregulated cytokines and chemokines, the two disease serotypes had their own subset of cytokine and chemokine dysregulation. EV-A71-associated HFMD cases displayed depressed expression of IL12p40, MCP-3, M-CSF, and IL-9 as well as augmented eotaxin. CV-A6-associated HFMD cases showed increased expression of IL-2Rα, GRO-α, IFN-α2, TRAIL, IL-1Rα, IL-10, and TNF-α.

Indeed, previous studies also presented HFMD cases of decreased levels of MIF, IL-8, MIP-1β, and M-CSF^29^, as well as increased levels of IL-18^42^, IL-5^36^, IP-10^42–44^, and eotaxin^29^. In addition, EV-A71 cases of increased levels of IL-18^32^ and IL-13^33^ have also been reported. Hence, these cytokines probably present generic cytokines implicated in HFMD cases. Some of the cytokines/chemokines including IL-8^32^, GM-CSF^30^, MCP-1^42,44^, IL-12p40^29^, and MCP-3^29^ were found to be dysregulated in opposite trends in other studies, thereby emphasising possible functional redundancies.

MIF, a pro-inflammatory cytokine of the immune system, is associated with disease severity and poor disease outcome^45^. Since HFMD is generally a mild and self-limiting disease and the patients from the Singapore cohort suffered from uncomplicated HFMD with full recoveries, MIF was expectedly decreased in most of them. IL-7 has also been reported to inhibit immunosuppressive Socs3 protein responsible for chronic virus infections^46^, possibly a key factor for a generally self-limiting disease. Cook *et al*. (1995) demonstrated that coxsackievirus-infected homozygous MIP-1α mutant mice suffered from less severe pneumonitis and were protected from virus-induced myocarditis even though there was delayed virus clearance in comparison to infected wildtypes^47^. Since MIP-1β shared 67% sequence identity with MIP-1α^48^, it was speculated that down-regulation of MIP-1β was also a measure to avert disease progression. IP-10 augmentation was not surprising since it was shown to inhibit CB3 viral replication^49^. MCP-3 has also been shown to inhibit viral replication as part of the anti-viral host defense mechanism^50^. M-CSF, shown to suppress CVB3 virus infection, reduce disease severity, and improve survival^51^, was reduced in EV-A71-associated HFMD cases.

IL-2 and GM-CSF were reduced perhaps to subvert host immunity. A recent publication by Felix *et al*. (2016) proposed the concept of virus-induced molecular mimicries in antagonising host-induced anti-viral cytokines (such as IL-2 and GM-CSF)^52^. The phenomenon of IL-8 reduction was not surprising as Dodd *et al*. (2001) showed how poliovirus, which belongs to the same family as enteroviruses, could limit IL-8 secretion during infection^53^. IL-15, a key regulator of anti-viral response during innate immunity^54^, was suppressed probably in the favor of the viruses. IL-9, reported to inhibit viral replication in CVB3-induced myocarditis^55^, was suppressed as well. IL-1Rα enhancement was also shown to be a virus’ evasion strategy^56,57^. FGF basic, which was reported to induce local infection to promote the healing process^58^, was significantly diminished. This might explain the higher probability of complications in EV-A71 infections^2–7^, as well as more persistency in the generally mild CV-A6 infections^59^ causing consequences such as onychomadesis as late onset manifestations. IL-16 was demonstrated to retard virus spread and control virus propagation^60^, and this could account for the late onset manifestations in the generally mild CV-A6 infections. TRAIL^61^ and IL-10^62^ has both pro- and anti-viral roles, hence it is unclear which role it serves in CV-A6-associated HFMD.

The suppression of IL-2, a Th1 cytokine, might possibly be a virus strategy to counter IL-2Rα increment. Indeed, IL-12, which is responsible for the induction of Th1 cytokines, was also diminished. IL-12 induction of Th1 cytokines was reported to be vital for anti-viral immunity and virus clearance^63,64^. These supported previous studies which reported implications of skewed Th2 cytokine response resembling the pathophysiology of allergic diseases in HFMD patients. The authors reasoned that the Th2-biased responses could be the fatal trigger for respiratory dysfunctions and failure^32^ or indicator of poor prognosis with higher fever and longer illness duration^65^.

Indeed, many cytokines and chemokines involved in a Th2 response or implicated in allergic diseases were dysregulated. IL-18, an imperative contributor of the generation of cytotoxic T lymphocytes against invading viruses^66^, was reported to be able to skew towards a Th2 response in the absence of IL-12 and IL-15^67^. β-NGF, produced by effector cells of allergic diseases, was amplified in HFMD patients as in allergic diseases such as asthma^68,69^ and allergic dermatitis^70,71^. SDF-1α, another factor implicated in chronic inflammatory skin diseases^72^ and asthma^73^, could also explain previous publications on HFMD similarity to allergic diseases. IL-5 is a pleiotropic Th2 cytokine commonly associated to asthma pathogenesis^74^. IL-13, also a Th2 cytokine, was implicated as an important contributor to virus-induced acute airway hyper-responsiveness and inflammation leading to asthma^75^. IL-7 was reported as a plausible contributor to airway inflammation in asthmatics^76^. Eotaxin, an eosinophil chemoattractant, was implicated in virus-induced airway inflammatory processes and disease exacerbations^77–80^. MCP-1, however, was surprisingly decreased since its augmentation was also previously linked to Th2 polarisation^81^. C-TACK, a predominantly cutaneous T cell selective chemokine^82^, was up-regulated. A skin-specific immune response was anticipated since HFMD is enanthematous and exanthematous in nature.

IFN-α2 and TNF-α have pyrogenic roles which may contribute to the febrile response commonly observed in patients with HFMD^83,84^. However the supposedly most potent endogenous pyrogens – IL-1α and IL-1β^85^ – were oddly diminished. Dinarello (2004) reported that infection-associated fever could be induced independent of pyrogenic cytokines (such as IL-1 and TNF) via TLR^86^. Another speculation was the virus’ strategy to regulate host temperature changes in order to persist in the host without much deterrence. Indeed, virus-shedding studies by Li *et al*.^87^ and Zhao *et al*.^88^ revealed EV-A71 could persist in the body for weeks even after the disease symptoms had subsided.

PDGF-BB induces proliferation through MAPK, JAK/STAT and PI3K pathways^89^. Wu *et al*. (2016) reported a positive correlation between cell proliferation and EV-A71 infection through a genome-wide RNAi screen, and revealed that many top hits were involved in cell proliferation or growth factor signalling pathways^90^.

In summary, it should be stressed that interpreting the overall outcome of the dynamic fluctuations of cytokine/chemokine cascade, instead of monitoring specific cytokine/chemokine alterations, would be more insightful to understanding the disease due to the functional redundancies of cytokines and chemokines^91^. This could also explain the irreproducibility of cytokine/chemokine dysregulation implicated in HFMD across different publications^26–39^. Our study also showed that the different cytokine/chemokine dysregulation triggered by EV-A71- and CV-A6-associated HFMD seemed to result in a similar clinical outcome. These observations could perhaps reflect the virus’ versatility in eliciting the same outcome through different channels. Hence, it is important to take into account the overall outcome(s) of the different combination of cytokine/chemokine dysregulation rather than a particular (set of) cytokine/chemokine dysregulation as disease biomarkers. From this study, it seemed that the viruses were striking an astute balance between exploitation of the host machinery for invasion and aversion of the host immunity for evasion.

First, we found that the dysregulation was seemingly triggered by the viruses in order to attain a balanced control of the host immune responses. In order for viruses to invade and propagate within the host, viruses need to modulate the host immune system such that they can take dominance over the host yet avoid over-stimulating the host immune system which will result in death. As such, viruses have to be able to strike a delicate balance to co-exist with and persist in the host, fighting off host anti-viral responses and restraining its invasion simultaneously. Next, a skewed Th2 response mirroring allergic diseases was presumably an attempt to avoid anti-viral immunity and virus clearance by Th1 cytokines^63,64^. In addition, the enanthematous and exanthematous nature of HFMD would naturally beget a skin-specific immune response. Since HFMD is a febrile illness, pyrogenic cytokines were also anticipated to be implicated. However, it was not surprising that cytokine-independent pyretic factors could still result in fever^86^. Lastly, an active cell mitotic status (G2/M phase of cell cycle) was postulated to promote viral replication in the proliferating cells. Moreover, mitotic progression could also initiate a switch from cap-dependent translation to internal-ribosome site-dependent translation necessary for enterovirus translation processes^90^.

While independent reports of EV-A71-induced ‘cytokine storm’, which describes a persistently over-stimulated immune response as a result of a positive feedback loop between cytokines and immune cells^92,93^, have been documented previously^37,94–96^, our study showed that the augmented cytokines in EV-A71-infected patients were also up-regulated in CV-A6-infected patients with the exception of eotaxin. Instead, EV-A71-infected patients had decreased sera levels of a subset of cytokines/chemokines not found to be significantly dysregulated in CV-A6-infected patients. In addition, CV-A6-infected patients had increased sera levels of another subset of cytokines/chemokines not found to be significantly dysregulated in EV-A71-infected patients. As previous studies have not explored CV-A6-associated cytokine/chemokine dysregulation, this pilot study showed for the first time the dynamics of this emerging HFMD causative agent in the host. Although CV-A6 usually causes self-limiting infections unlike neurotrophic EV-A71, its persistence causing consequences such as onychomadesis as late onset manifestations remained puzzling. These findings showed that CV-A6 is capable of eliciting greater (or at least the same) cytokine/chemokine dysregulation than (as) EV-A71, thereby highlighting a need to explore serotype-specific cytokine/chemokine dysregulation in HFMD patients to understand the clinical presentations associated with the respective etiological agents.

For example, onychomadesis was reported in young children who did not receive treatment from autosomal recessive loss-of-function mutations in *IL*1*RN* gene resulting in IL-1Ra deficiency. Interestingly, CV-A6-infected patients were found to have diminished levels of IL-1α and IL-1β as well as augmented levels of IL-1Ra^97^. Since onychomadesis is a late onset manifestation observed in CV-A6-associated HFMD cases, it was speculated that the over-compensation of cytokine/chemokine dysregulation might have triggered this. Other than CV-A6-associated HFMD, patients recovering from toxic shock syndrome also experienced desquamation one to three weeks after onset as well as nail shedding and Beau’s lines after recovery^98^. Cytokines such as TNF-α, IL-1, IL-6, IL-2, and IFN-γ were amplified in these patients. The expression of TNF-α was also increased in CV-A6-infected patients. Psoriasis lesions were found to have increased levels of cytokines including TNF-α and decreased levels of cytokines including IL-1 and IL-10. However, Saulite and colleagues found augmented IL-10 in psoriasis-affected nail beds, suggesting that IL-10 could specifically be linked to nail changes^99^. Coincidentally, IL-10 was also exhibited at high levels in CV-A6-associated HFMD cases. It would be interesting to study the implications of differential cytokine/chemokine dysregulation between EV-A71 and CV-A6 by exploring the influence(s) of these cytokines and/or chemokines on the pathogenesis of the two viruses.

Despite a small sample size, this is the first study on the cytokine/chemokine profiles of the HFMD patients from the Singapore and Malaysia cohort. In particular, we showed that the Singapore and Malaysia cohorts had differential cytokine/chemokine dysregulation, even when infected with the same causative serotype (i.e. EV-A71). Further analyses showed that differences were observed even when comparison was made between different periods of recruitment within the same cohort. This showed that causative EV-A71 genotype played a contributing role in the differential dysregulation. Comparative analyses between the two major causative serotypes isolated from the Singapore cohort showed that causative serotype played a contributing role in the differential dysregulation as well. We showed for the first time the similarity and dissimilarity in the cytokine/chemokine dysregulation between EV-A71- and CV-A6-infected HFMD patients. As samples were obtained from consented patients upon hospitalisation, it was not possible to pre-determine the stage of infection the patients were in during sample collection. Hence, the differential profiles could also be partly influenced by the different stages of infection. Nevertheless, we hypothesised that the viruses were capable of flexible fine-tuning of the plethora of cytokines/chemokines towards a similar outcome. Further studies remain to explore and understand the intricate network of cytokines and chemokines triggered or attenuated by the virus or host to beget a particular clinical outcome.

## Materials and Methods

### Subject recruitment, sample collection and disease status verification

This study was approved by and carried out under the guidelines of the Ethics Committee of the SingHealth Centralised Institutional Review Board (CIRB; CIRB reference number: 2012/448/E) for the recruitment from KK Women’s and Children’s Hospital, Singapore, as well as the Medical Ethics Committee (reference number: 872.7) and the Medical Research and Ethics Committee of the Ministry of Health, Malaysia (reference number: NMRR-12-1038-13816) for the recruitment from the University of Malaya Medical Center, Malaysia. Informed consent was obtained from the parents/guardians of all the recruited subjects.

In this study, the patients were evaluated by clinicians using a clinical case definition of fever, oral ulcers, and skin lesions on the palms and soles for HFMD. The clinically-defined HFMD cases were confirmed using at least one of the laboratory tests, including viral genotyping, virus isolation and genotyping, as well as anti-EV-A71 IgM antibody detection. Only laboratory-confirmed cases were included in this study. Serum samples from patients diagnosed with HFMD and healthy volunteers were collected for cytokine and chemokine profiling.

### Human Cytokine and Chemokine Bio-Plex Assays

The patient and healthy sera were assayed using the Bio-Plex Pro^TM^ human cytokine 21-plex assay kit (Bio-Rad #MF0005KMII) and Bio-Plex Pro^TM^ human cytokine 27-plex assay kit (Bio-Rad #M500KCAF0Y) according to the manufacturer’s instructions. The raw data was processed using Bio-Plex Manager software version 6.1 (Bio-Rad Laboratories, USA). Cytokines with out-of-range (OOR) readings that were below its detection limits were recorded as 0.1 pg/ml for subsequent statistical analyses which involved log transformation of data.

### Statistical Analyses

All statistical analyses were performed using GraphPad Prism version 4.0 (GraphPad software, USA), using Mann-Whitney or Kruskal-Wallis non-parametric tests (without Gaussian distribution assumption). Kruskal-Wallis non-parametric tests were coupled with Dunns post-test to compare all pairs of groups. All p-values were automatically adjusted by the program to account for false discovery rates associated with multiple comparisons. (Appropriately-adjusted) p-values below 0.05 were considered to be statistically significant.

## Electronic supplementary material

Supplementary Information
