# Supplementary material for: Cytokine and Chemokine Profiling in Patients with Hand, Foot and Mouth Disease in Singapore and Malaysia
Source: Sci Rep. 2018 Mar 6;8:4087. doi: 10.1038/s41598-018-22379-6 (PMC5840398; doi:10.1038/s41598-018-22379-6)

Cytokine and Chemokine Profiling in Patients with Hand, Foot and Mouth Disease in Singapore and Malaysia.

Fiona Mei Shan Teo[1], Min Nyo[2] Anng Anng Wong[3], Natalie Woon Hui Tan[3], Mia Tuang Koh[4], Yoke Fun Chan[5], Chia Yin Chong[3] and Justin Jang Hann Chu[1, 2*]

[1] Collaborative and Translation Unit for HFMD, Institute of Molecular and Cell Biology, Agency for Science, Technology and Research (A*STAR), Singapore.

[2] Laboratory of Molecular RNA Virology and Antiviral Strategies, Department of Microbiology and Immunology, Yong Loo Lin School of Medicine, National University of Singapore, Singapore.

[3] Infectious Disease Service, Department of Pediatrics, KK Women's and Children's Hospital, Singapore.

[4] Department of Pediatrics, Faculty of Medicine, University of Malaya, Kuala Lumpur, Malaysia.

[5] Department of Medical Microbiology, Faculty of Medicine, University of Malaya, Kuala Lumpur, Malaysia.

Correspondence: Justin Jang Hann Chu, Institute of Molecular and Cell Biology, Proteos, 61 Biopolis Drive, Singapore. Email: jhchu@imcb.a-star.edu.sg

Supporting Information

Supplementary Table S1 | Summarised table of the dysregulated cytokines/chemokines in the respective cohorts/groups. p value < 0.05, p value < 0.01

	Singapore EV-A71 vs Malaysia EV-A71	Msia2000 EV-A71 vs Msia2012 EV-A71	SgCV-A6 vs Healthy	SgEV-A71 vs Healthy	SgEV-A71 vs SgCV-A6
IL-1α					
IL-2Rα					
IL-3					
IL-12p40					
IL-16					
IL-18					
CTACK					
GROα					
HGF					
IFN-α2					
LIF					
MCP-3					
M-CSF					
MIF					
MIG					
β-NGF					
SCF					
SCGF-β					
SDF-1α					
TNF-β					
TRAIL					
IL-1β					
IL-1Rα					
IL-2					
IL-4					
IL-5					
IL-6					
IL-7					
IL-8					
IL-9					
IL-10					
IL-12p70					
IL-13					
IL-15					
IL-17A					
Eotaxin					
FGF basic					

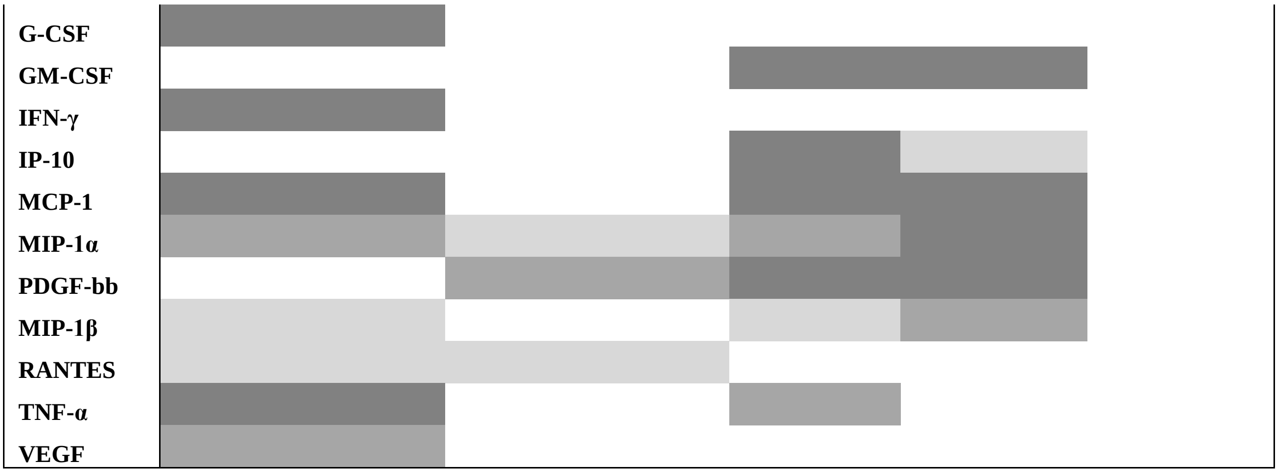

Legend: The shades of grey represents the significance of statistical tests. White/blank indicates no statistical differences. The lightest shade of grey indicates p < 0.05. The mid shade of grey indicates p < 0.01. The darkest shade of grey indicates p < 0.001

Supplementary Figure S2 | The expression profiles of HFMD patients recruited from Malaysia in 2000 and those recruited in 2012 showed significant differences in 13 out of the panel of 48 cytokines and chemokines assayed. The statistical analyses were performed using GraphPad Prism version 4.0 (GraphPad software, USA), using Mann-Whitney non-parametric tests (without Gaussian distribution assumption). * p value < 0.05, ** p value < 0.01, *** p value < 0.001.

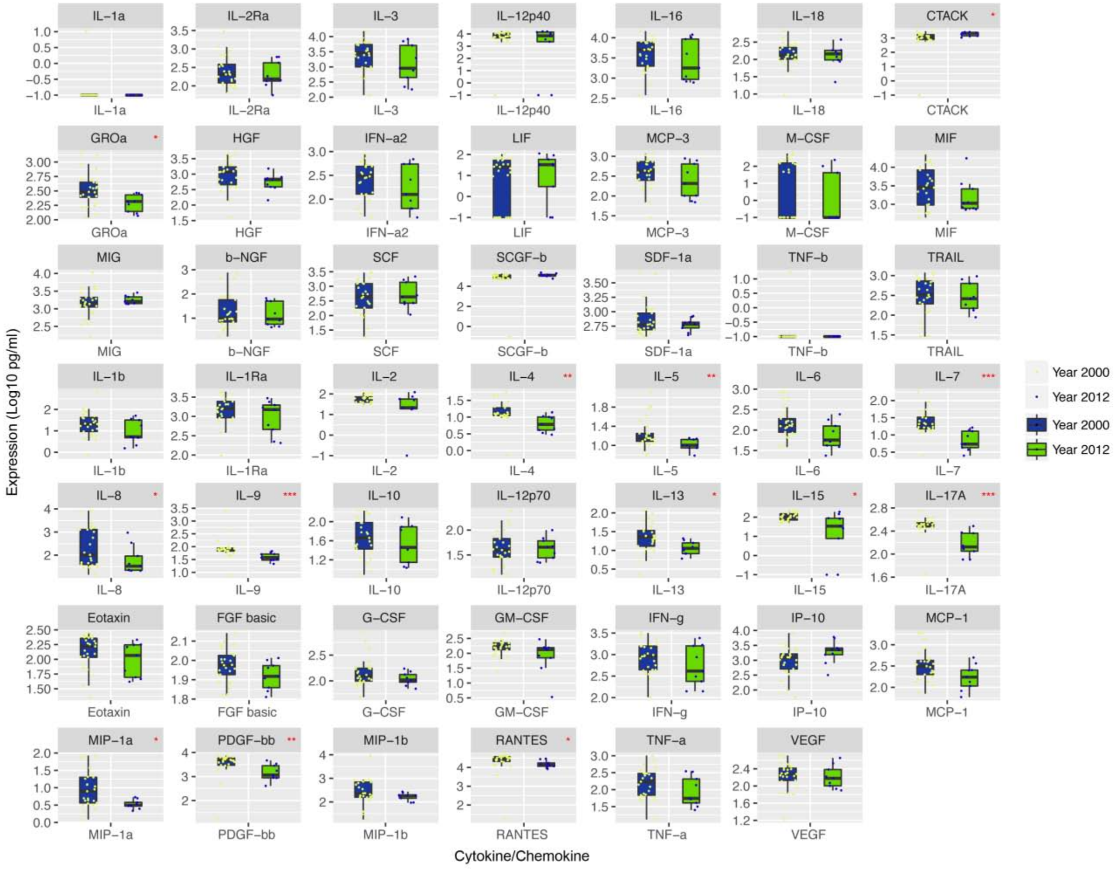

Supplement: Supplementary file 1 — Supplementary Information [file 41598_2018_22379_MOESM1_ESM.pdf]
